# Kinetic Model with Feedback Cycle for Age-Dependent Amyloid Beta Accumulation in Mice

**DOI:** 10.3390/ijms26188803

**Published:** 2025-09-10

**Authors:** Vivian Tyng, Michael E. Kellman

**Affiliations:** 1 Department of Chemistry and Biochemistry, University of Oregon, Eugene, OR 97403, USA; tyng@uoregon.edu; 2 Materials Science Institute, University of Oregon, Eugene, OR 97403, USA; 3 Institute for Fundamental Science, University of Oregon, Eugene, OR 97403, USA

**Keywords:** amyloid beta, positive feedback, vicious cycle, kinetic modeling, Alzheimer’s disease, combination interventions

## Abstract

Amyloid beta (Aβ) is believed to play a key role in Alzheimer’s disease (AD), whose causes, progression, diagnosis, and treatment nonetheless remain poorly understood despite decades of research. Recent studies suggest that Aβ in its various forms participates in multiple mutual feedback loops (“vicious cycles”) including tauopathy, oxidative stress, inflammation, calcium dysregulation, excitotoxicity, and probably many others, eventually leading to neurodegeneration and cognitive decline. Here, as an initial quantitative step toward modeling this vast complexity, we explore a simple kinetic model of a coupled feedback vicious cycle for Aβ buildup based on literature data for Tg2576 mice. The model is used to examine the efficacy of various hypothetical therapeutic approaches, either singly or in combination, to mitigate Aβ buildup. While our computational results support the possible efficacy of combination interventions, they also suggest caution, inasmuch as clear synergy is not found. This kinetic approach highlights the essential importance of the vicious cycle of positive feedback in a quantitative model.

## 1. Introduction

Since Alois Alzheimer reported the first case of Alzheimer’s Disease (AD) almost 120 years ago, a vast amount of research has been conducted on AD, including its pathogenesis, diagnosis, course prediction, and treatment options. Amyloid beta (Aβ) is a major pathological feature of AD, and its crucial role in the disease is generally accepted [1]. The amyloid cascade hypothesis [2] further proposes Aβ as a key causal factor in AD. However, development of drugs targeting Aβ has encountered enormous challenges. Only in the last few years have a few drug candidates shown promising results in Phase III clinical trials and were finally approved by the FDA [3]. Some of the challenges are due to the nature of Aβ (an intrinsically unstructured protein that forms different aggregates), the difficulty of measuring Aβ levels in living human subjects, and a lack of suitable animal models. Another main obstacle is that the accumulation of Aβ (regardless of form) is probably coupled to many positive feedback loops [4,5]. Proposed examples include tauopathy (hyperphosphorylated tau protein, another hallmark of AD) [6,7], oxidative stress [8], neuroinflammation [9], vascular dysfunction [10], and abnormal glucose metabolism [11]. Nevertheless, feedbacks proposed so far for the pathogenesis and progression of AD are heterogeneous and operate at different levels from molecular to systemic, and the exact mechanisms are often difficult to isolate.

In this paper, we seek to begin to model Aβ accumulation in a quantitative way. We construct a kinetic model of Aβ production using data from a mouse model of AD studied by Parkinson et al., in which runaway Aβ production is observed, due to a positive feedback or “vicious cycle” mechanism [12]. This model was developed as described in the remainder of this Introduction. This use of a kinetic model has grown in part out of a previous model based on ordinary differential equations (ODEs) to describe translational autoregulation, albeit using a negative instead of positive feedback term [13].

In their empirical study, while investigating the pharmacokinetics of an AD drug candidate, Parkinson et al. measured the levels of soluble and insoluble Aβ in the brains of Tg2576 transgenic mice as a function of their age [12]. Although animal models do not fully replicate the Aβ accumulation process (much less the AD progression) in humans [14], the trend of the mice data resembles that of humans found in both longitudinal follow-up [15] and post-mortem analysis of a large population [16]. The overall time course is sigmoidal: first a considerable latency period in which the soluble and insoluble Aβ levels are low, a rapid acceleration in the middle, and finally a plateau at old age with notably higher levels (by a factor of 100 or more compared to the initial value).

Believing that this dataset offers a chance to model Aβ accumulation in a semi-quantitative way, here we fit it using a kinetic model with a positive feedback loop. During the fitting, we confirmed that it is necessary to have a very large feedback to account for the massive increase in Aβ over the mice’s lifetime; whereas pure linear kinetics fails completely. Using the obtained fit, we consider various intervention strategies to inhibit Aβ accumulation.

It must be emphasized the mice data are far from providing a definitive understanding of Aβ dynamics in humans, especially considering the simple framework used here. Our goal here is as much to raise necessary questions as to provide even tentative answers. The present model serves as a possible basis for constructing a model of humans, in which basic aspects of nonlinear kinetics can be used to explore presently unknown aspects of Aβ kinetics.

## 2. Materials and Methods

### 2.1. System and Kinetic Data

Parkinson et al. [12] measured the soluble and insoluble Aβ40 and Aβ42 concentrations in the whole brains of Tg2576 transgenic mice over their lifetime (up to 24 and 26 months (mo) for Aβ40 and Aβ42, respectively). These mice, which have long been used as an animal model for AD, overexpress a mutated version of the human amyloid precursor protein (APP) and exhibit Aβ accumulation but no tau pathology and only minimal neuronal loss or cell death. Indeed, the data in Ref. [12] show a dramatic, lengthy progression of Aβ levels: first a long latency period, then a dramatic rise in the middle (in which the soluble and insoluble fractions increased by a factor of 100 and 1000, respectively), and finally a saturated plateau at later times. Those authors fitted the overall Aβ accumulation by logistic functions. Here, we focus on the soluble Aβ42 because (1) Aβ42 is the main component of insoluble amyloid plaques [17] and (2) kinetics of soluble Aβ is better characterized than the insoluble form. For the soluble Aβ42, its logistic formula is

(1) $$\begin{array}{c} A_{logistic}\left(t\right)=34+\frac{2240}{1+e^{-5.9ln(10)log(t/18)}} \end{array}$$

where *A* is the Aβ concentration in pg/mg, and *t* is the time in months. In the kinetic models with coupled feedbacks that we construct below, the A*_logistic_* values calculated from Equation (1) at *t* = 1, 2, …, 27 will be used as the target “data”.

### 2.2. Kinetic Model

#### 2.2.1. Considerations on Kinetics and Modeling of Mouse Data

Before proceeding with a concrete kinetic model, we consider some well-accepted information on the kinetics of Aβ. (1) The turnover time of amyloid monomers (i.e., half-life from synthesis to degradation) is on the order of hours, which is much shorter than the disease progression (months in mice and years in humans) [18]. Therefore, the monomer can be considered in quasi-steady state all the time. (2) According to accumulated animal and human data, the dramatic accumulation of Aβ during disease progression is probably driven by some type of positive feedback process, instead of just linear processes of enhanced production or inhibited clearance [4,19,20,21]. For more background about these vicious cycles, see the Introduction section.

First of all, we demonstrate that a simple linear model with only steady production of Aβ and its first-order degradation does not reproduce the sigmoidal pattern at all. Specifically, if we take

(2) $$\begin{array}{c} \frac{dA(t)}{dt}=k_{1}-k_{2}A\left(t\right), \end{array}$$

the solution $A\left(t\right)=k_{1}/k_{2}+\left(A\left(1\right)-k_{1}/k_{2}\right)e^{-k_{2}\left(t-1\right)}$ is not sigmoidal (characterized by slow–fast–slow growth rates) for any choices of $k_{1},k_{2}$, because the growth rate of A(t) is the fastest at t=1 and decreases continuously in time. So, a nonlinear model is needed.

#### 2.2.2. Kinetic Model with Nonlinear Feedback, and Fitting to the Model

**Figure 1:**
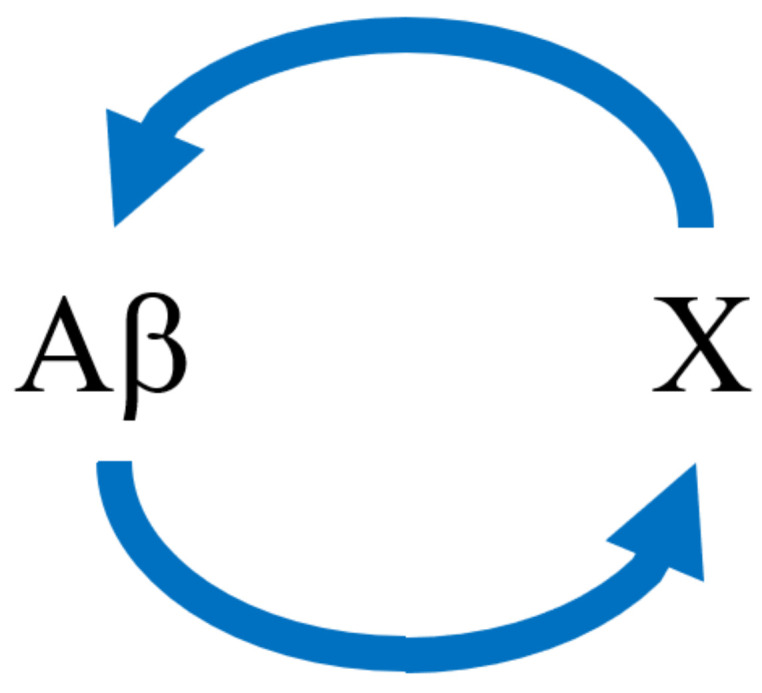
Schematic of the dual positive vicious cycle feedback model for Aβ and *X*. Note that the arrows represent stimulation (i.e., positive feedback) instead of chemical conversion.

We next suppose that Aβ and an abstract variable *X* (which could represent tauopathy or oxidative stress, for example) stimulate each other’s production, forming nonlinear feedback cycles. A diagram of the scheme is shown in Figure 1.

In this model, Aβ (A(t) in Equation (3), units: pg of Aβ per mg of wet brain tissue) enhances the production of X(t) in Equation (4) (units: dimensionless, since it can represent various biochemical processes such as tauopathy or oxidative stress), and vice versa. Both *A* and *X* degrade with first-order kinetics. Specifically, we have

(3) $$\begin{array}{c} \dot{A}\left(t\right)=\frac{V_{1}X{\left(t\right)}^{2}}{{\left(K_{1}\right)}^{2}+X{\left(t\right)}^{2}}-k_{2}A\left(t\right) \end{array}$$

(4) $$\begin{array}{c} \dot{X}\left(t\right)=\frac{V_{1}^{\prime }A{\left(t\right)}^{2}}{{\left(K_{1}^{\prime }\right)}^{2}+A{\left(t\right)}^{2}}-k_{2}^{\prime }X\left(t\right) \end{array}$$

The first term on the right hand side of Equation (3) and Equation (4) is a Hill-type function that reflects the mutual positive feedback between *A* and *X* on each other’s production. $V_{1}$ and $V_{1}^{\prime }$ describe the magnitude of the feedback, namely, the plateau heights of *A* and *X*, respectively. $K_{1}$ and $K_{1}^{\prime }$ reflect the sensitivity of the feedback, namely, how soon the Hill function reaches its half maximum [22]. The Hill coefficient *n* was fixed at 2, which is the smallest integer that produces a sigmoidal trend. Because our model *phenomenologically* describes the accumulation of Aβ at the organism level and a non-specified process *X*, we will refrain from elaborating on the physiological meaning of these parameters, even though more concrete interpretations of the Hill equation have been discussed in the literature for specific systems such as cooperative binding, enzymatic activity, and pharmacology.

During fitting, parameters in the kinetic model in Equations (3) and (4) were varied to minimize the summed log error in *A* ($\sum |log(A_{pred}\left(i\right)/A_{logistic}\left(i\right))|$, i=1−27), where $A_{pred}\left(i\right)$ is the *A* value predicted by integrating the kinetic Equations (3) and (4). $A_{logistic}\left(i\right)$ are the values given by the logistic fit in [12] and reproduced here as Equation (1).

**Figure 2:**
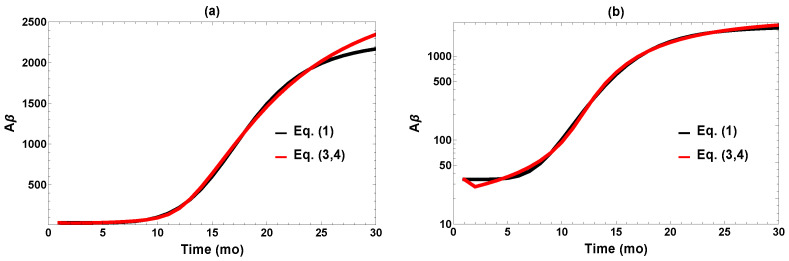
Comparison of fit to the target data (Equation (1)) plotted on (**a**) linear and (**b**) logarithm scales.

To reduce the number of parameters, we fixed $k_{2}$ at 450 (unit: mo^−1^), the value postulated in [12], which comes from the established turnover time of soluble amyloid in mice (1.1 h, compared to 8 h in humans). The initial value A(1) = 34 pg/mg is the baseline level obtained experimentally in [12]. For the non-specified *X*, the initial value was arbitrarily fixed at X(1)=1; our results do not depend significantly on this choice. The fitted parameter values are provided in Table 1, and a comparison of the fit and the target $A_{pred}\left(i\right)$ values are plotted in Figure 2 on both linear and logarithmic scales. It is evident that fitting to the feedback model works well within the limitations of available data.

### 2.3. The Question of Bistability

De Caluwé and Dupont proposed a kinetic model with highly nonlinear behavior by assuming mutual positive feedback between Aβ accumulation and growing intracellular calcium ion concentration [23]. Our vicious cycle model is in some respects similar to their model, but there are significant differences in the kinetic equations, aims, and presumptions. Their model displays striking features of bistability that play an essential role in their conception of the AD process. They suppose a “jump” from a healthy to a diseased state, associated with a critical point of bistability in their kinetic model. A similar idea of bistability is outlined schematically by Burlando et al. [24]. Does such a picture pertain to the model considered here? This turns out not to be the case, and it is worthwhile to consider why.

The bistable behavior in the model of [23] is suggestive of certain actual features of the AD process in humans. In one study of human subjects not pre-selected for their Aβ status, the brain amyloid levels measured post mortem appear to be clustered around a healthy state and a diseased state [25]. In another longitudinal study of living subjects, the transition from Aβ-negative to Aβ-positive (measured by positron emission tomography (PET)) over the years is generally irreversible [26]. These results are at least consistent with the bistable model of Ref. [23], in which a jump from a healthy to a diseased steady state occurs when a certain bifurcation point of bistability sets in as the Aβ system evolves in time. While highly suggestive, at present the bistable jump model is schematic and speculative, since quantitative time-course data for the brains of living humans are multidimensional, the measurement technologies are far from mature, and the data have intrinsic wide distribution.

We initially were very open to the possibility that our mouse model might show behavior like the jump model of [23]. However, we have not found evidence that bistability plays such a role. The reason is that bistability requires an interaction or competition between linear and feedback processes, whereas the feedback term produced by our fitting to the mice data is massive and overwhelms the linear terms. This moves the “jump-off” bifurcation to an Aβ level far below anything relevant at any point in the mouse life trajectory. Instead, the vicious cycle gradually but inexorably moves the mouse on a lifelong path of disease. This could be called a “drift” picture, rather than a bistable “jump.” It is worth noting that we find this also in variants of our model, e.g., one in which we introduce a linear basal Aβ production term into our kinetic scheme of Equations (3) and (4). There is simply not presently a role for the jump, based on the data. The model of Ref. [23] was specifically constructed to produce a jump as proof of concept, with parameters not tied to quantitative data. It would be of great interest in the future to have data to tell if the human disease process is more like the jump idea of Ref. [23].

## 3. Results and Discussion

### 3.1. Intervention in the Disease Process

Now we use the model developed above to think about strategies for intervening in the mouse disease process, with a view toward eventual intervention in humans. Despite decades of extensive search, a safe and effective way to treat AD or even reverse its progression remains elusive. It is now widely thought that the amyloid hypothesis by itself is too simple [27], and some feedbacks with other species should be considered, as explained in the Introduction section. Consistent with this point of view, our model incorporates a feedback loop involving both Aβ and the hypothesized factor “X” to explore possible interventions. Another current line of thought is that single interventions may need to be replaced by combined interventions. We explore this in the context of the model. In the following, we first review tested drug candidates in relation to the principles of the model, then simulate the effect of intervening to vary the different model parameters on the time course of Aβ accumulation. We evaluate the advantages from combinational interventions targeting both linear and nonlinear terms in the model. In the future, we hope to build similar models for humans to explore clinical interventions.

### 3.2. Clinical Strategies of Intervention

Our model is based on somewhat abstract principles but relatable to strategies used in actual drug development. Due to the many unique challenges in developing AD drugs, no symptomatic or disease-modifying treatments were approved by the US FDA between 2003 and 2020 out of hundreds of substances tested in clinical trials. Only the last four years saw the approval of three monoclonal antibodies: aducanumab (approved in 2021 and removed from the market in 2024), lecanemab (approved in 2023), and donanemab (approved in 2024). Among disease-modifying drug candidates in previous or ongoing clinical trials, the following have hypothetical mechanisms relevant to our kinetic model for Aβ:

1. Simply removing Aβ corresponds to increasing $k_{2}$ in our model. Quite a few monoclonal antibodies bind to various forms of Aβ and facilitate their degradation: aducanumab (oligomers and fibrils), gantenerumab (oligomers and fibrils), lecanemab (protofibrils), solanezumab (monomers), crenezumab (mainly oligomers, and also monomers and fibrils to a lesser extent), and donanemab (mature plaques) [28]. Although effective Aβ removal was observed in phase III clinical trials through PET imaging, whether it translates to meaningful cognitive benefit remains hotly debated [29,30].

2. The processing of APP involves various secretases. The Aβ peptides (such as Aβ40 and Aβ42) are generated through the sequential action of β- and γ-secretases, while α-secretase cleaves APP within the Aβ sequence to form other products. Therefore, the amount of Aβ may be lowered by either inhibiting β/γ-secretases or enhancing the activity of α-secretase. Several β-secretase inhibitors have undergone clinical trials, albeit with disappointing results, especially in terms of side effects. The drug candidate APH-1105 also enhances α-secretase and thus suppresses Aβ production by competition. In our model, the effect of β-secretase inhibition, for example, would be reflected by reducing the parameter $V_{1}$.

3. As mentioned earlier, many biomolecular processes are likely involved in a vicious cycle with Aβ accumulation. These correspond to species X in our kinetic model. Many drug candidates target tau, such as TRx0237 (inhibits tau aggregation), ABBV-8E12 and BIIB092 (monoclonal antibodies against extracellular aggregated tau), and BIIB080 (RNA inhibitor against tau expression). Drug candidates targeting other vicious cycles include ALZ-OP1 (anti-inflammation), metformin (targeting insulin insensitivity), and nilvadipine (targeting calcium dysregulation). In our model, the effect of these drugs might be simulated by reducing $V_{1}^{\prime }$, increasing $K_{1}$, and/or increasing $k_{2}^{\prime }$.

### 3.3. Effects of Single Interventions

Now, we discuss possible interventions implemented by changing the parameters in the model. Of the eight parameters in Table 1, A(1) and X(1) were fixed during fitting and so are not suitable candidates for intervention. Reducing these initial values would not help retard disease progression anyway, considering the massive buildup later. Overall, the accumulation of Aβ could be ameliorated by (1) increasing the parameters $k_{2}$ and/or $k_{2}^{\prime }$, i.e., accelerating first-order removal of *A* and *X*, (2) reducing $V_{1}$ and/or $V_{1}^{\prime }$, i.e., reducing the magnitude of feedbacks, and (3) increasing $K_{1}$ and $K_{1}^{\prime }$ to delay the onset time of feedback without reducing the saturation levels [31]. However, due to a lack of understanding at present of the physiological meaning of $K_{1}$ and $K_{1}^{\prime }$, these two parameters are excluded from discussion from this point on. This leaves the four parameters $k_{2}$, $k_{2}^{\prime }$, $V_{1}$, and $V_{1}^{\prime }$ as suitable candidates for intervention. We will first consider single interventions by varying these four parameters separately.

For each intervention, we define the dose as

(5) $$\begin{array}{c} D=|1-P/P_{0}| \end{array}$$

where $P_{0}$ and *P* are the kinetic parameter value without intervention (c.f. Table 1) and with intervention, respectively. This definition is valid not only for suppressing the production of a biomolecule but also for accelerating its removal. In both cases, D=0 means no intervention, and D=1 means doubling the parameter value (for $k_{2}$ and $k_{2}^{\prime }$) or setting the parameter value to 0 (for $V_{1}$ and $V_{1}^{\prime }$). Note that with this definition, D>1 is also possible for the removal parameters $k_{2}$ and $k_{2}^{\prime }$, while that of the production parameters $V_{1}$ and $V_{1}^{\prime }$ is capped at 1.

**Figure 3:**
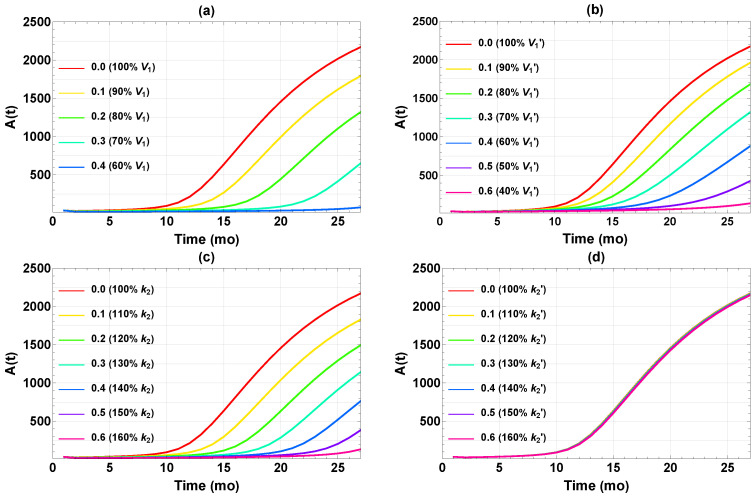
Time courses of A(t) after implementing different single interventions starting at *t* = 1 mo in (**a**) $V_{1}$, (**b**) $V_{1}^{\prime }$, (**c**) $k_{2}$, and (**d**) $k_{2}^{\prime }$. The legends show (left) the doses and (right) the corresponding changes in parameter value relative to those in Table 1.

Figure 3 displays the outcome of the four single interventions implemented permanently at the age of 1 mo (when the mice reach adulthood). Not surprisingly, the Aβ level depends on the dosage for each intervention. At reduced $V_{1}$ or $V_{1}^{\prime }$, Aβ generally still accumulates albeit at slower rates. In the rest of the paper, we choose the somewhat arbitrary value of A(27)≤100 as the target for “successful intervention,” which is rather stringent because this is less than 1/20 of the final Aβ level without treatment. For interventions via $V_{1}$, $V_{1}^{\prime }$, and $k_{2}$, a change of −39%, −64%, and +63% (dose = 0.39, 0.64, and 0.63, respectively, according to Equation (5)) would reach this target. Meanwhile, a comparable change in $k_{2}^{\prime }$ has no noticeable effect on the Aβ buildup (Figure 3d). We see that effective results could be achieved in three of the four single interventions when implemented aggressively, maintaining a high dose for the whole adult life of the mouse. The required minimum dosage is quite sensitive to the specific intervention.

**Figure 4:**
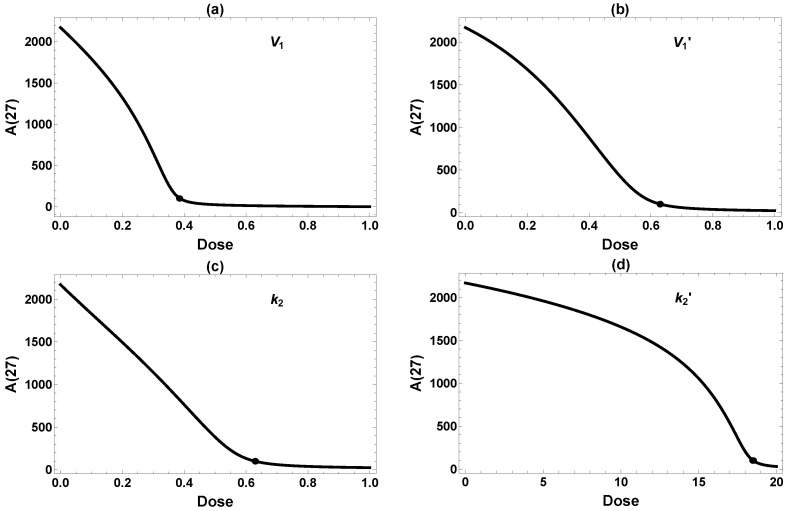
Dose-dependent effects on A(27) from single interventions in (**a**) $V_{1}$, (**b**) $V_{1}^{\prime }$, (**c**) $k_{2}$, and (**d**) $k_{2}^{\prime }$. All interventions are implemented permanently starting at 1 mo. The dots label the minimum dose required to attain the desired reduction of A(27)≤ 100. Because the model is less sensitive to changes in $k_{2}^{\prime }$, a very high dose is required to achieve meaningful amyloid reduction in (**d**).

Figure 4 further illustrates the effect of the single intervention dosage on the final Aβ level (A(27)). There seems to be a fairly distinct “threshold” dose for each intervention, above which A(27) falls below the target level of 100 and remains low. This is not exactly surprising, since these interventions are designed to reduce A(t), which is also constrained to be positive. Therefore, A(27) falls upon increasing the dosage, even in the less effective case of $k_{2}^{\prime }$ in Figure 4d, until the reduction approaches the baseline of zero. Still, the thresholds are rather sharp, which has implications for efficacy of interventions. It is essential to have the dose high enough to achieve the target Aβ level of 100. In summary, three of four single interventions work reasonably well if they can be implemented at a high enough dose at the very early age of one month.

**Figure 5:**
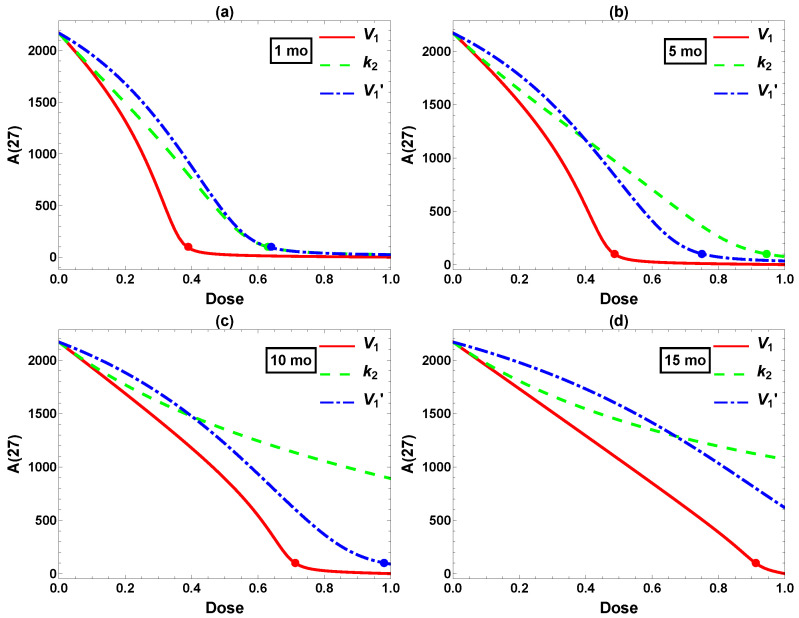
Comparison of interventions in $V_{1}$, $k_{2}$, and $V_{1}^{\prime }$ starting at (**a**) 1, (**b**) 5, (**c**) 10, and (**d**) 15 mo. *x*-axis: intervention dose defined in Equation (5). *y*-axis: Aβ level at 27 mo. The dots label the minimum dose required to attain the desired reduction A(27)≤ 100.

Next, we compared the three single interventions (excluding the ineffective $k_{2}^{\prime }$) starting at different ages. The corresponding A(t) data are plotted in Figure 5. Clearly, the final Aβ level depends on the intervention intensity as well as the timing. A 50% reduction in $V_{1}$ (red) is highly effective when starting at t=1, but successful intervention starting at *t* = 15 would require a reduction of more than 90%. Similar trends are observed for $k_{2}$ and $V_{1}^{\prime }$. This agrees with the experimental and clinical observations that earlier intervention tends to produce more pronounced effects, although other factors such as side effects and cost–benefit would have to be considered in practice. In the case of Aβ reduction in Tg2576 mice, we conclude that effective intervention would best be implemented at moderate dose and before 5 mo, with effective later intervention requiring a much higher, perhaps unrealistic dose. These are rather stringent requirements—starting at 5 mo less so than 1 mo, but the treatment course still spans most of the lifetime of the mouse. We next turn to the usefulness of combining multiple interventions.

### 3.4. Combination Interventions

We have seen that effective single interventions have stringent demands, requiring treatment beginning early in the mouse’s lifespan, and continuing indefinitely. We now consider to what extent multiple interventions offer hope of more effective treatment strategies within the dynamical model. Multi-pronged approaches to treat AD have been advocated for a long time, although only a handful of combined clinical trials have been reported. It is hoped that new trial designs and updated regulatory guidelines would make such trials easier to implement [32].

Having examined the effect of changing one model parameter at a time, we now combine two parameter changes as interventions. Table 2 shows the dose required to achieve the target A(27)≤100 when using either one intervention (diagonal) or a combination of two interventions at the same dose (off-diagonal). (All interventions are implemented permanently after t=1). A smaller minimum dose means more effective treatment. As seen from the table, $V_{1}$ monotherapy and all three combinations achieved reasonable efficacy, with a required minimum dose of 0.24–0.39. In contrast, $k_{2}$ and $V_{1}^{\prime }$ monotherapies have a minimum required dose of 0.63 or higher (as shown earlier in Figure 3). Clearly, the more effective (single or double) interventions require dampening of the feedback via $V_{1}$ and/or $V_{1}^{\prime }$, instead of simply accelerating the first-order degradation ($k_{2}$, $k_{2}^{\prime }$). The trick will be to figure out how to intervene on $V_{1}$ in the feedback vicious cycle—this seems much less clear than intervening in the linear terms.

**Figure 6:**
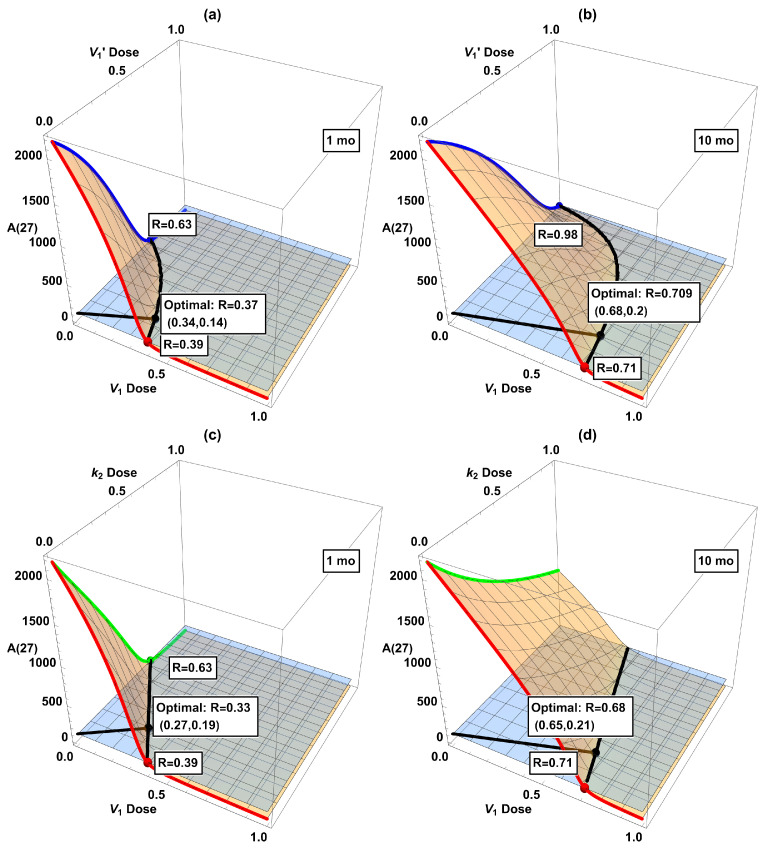

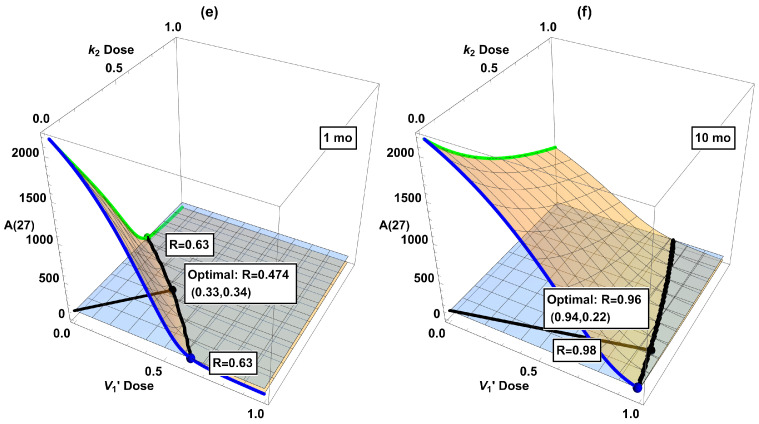
Dose-response surfaces (orange) when combining two interventions: (**a**) $V_{1}$ + $V_{1}^{\prime }$ starting at 1 mo, (**b**) $V_{1}$ + $V_{1}^{\prime }$ starting at 10 mo, (**c**) $V_{1}$ + $k_{2}$ starting at 1 mo, (**d**) $V_{1}$ + $k_{2}$ starting at 10 mo, (**e**) $V_{1}^{\prime }$ + $k_{2}$ starting at 1 mo, and (**f**) $V_{1}^{\prime }$ + $k_{2}$ starting at 10 mo. The red, green, and blue curves (as well as the dots in matching color) correspond to single treatments in Figure 5. The horizontal plane (light blue) corresponds to the desired reduction of A(27)=100, and its intersection with the dose–response surface (black curve) indicates the minimum dose combination required to attain A(27)≤ 100. The black dot labels the point with the shortest Cartesian distance from the origin to the black curve of desired reduction (“optimal combination”).

In Table 2, we combined two interventions at the same strength. However, the doses of individual drugs in actual combinational therapy should be adjusted according to their efficacy and toxicity. Hence, we have further considered two interventions in all possible dose combinations. The results are displayed as 3D plots in Figure 6. Intersections of the 3D surface with the side walls in the plot correspond to single interventions ($V_{1}$, $V_{1}^{\prime }$, or $k_{2}$). The black curve in each panel is the intersection in the horizontal plane of the dose–response surface (orange) and the desired threshold A(27)=100 (light blue). The shortest Cartesian distance *R* between this curve and the origin, indicated by the black line segment from the origin to the black dot, might be taken as the optimal combination. While certainly being somewhat arbitrary, this definition of the optimal dose seems not unreasonable. For present purposes the exact definition should not alter the qualitative conclusions we will present below. (In considering optimality, we ignore the issue of drug toxicity due to the highly abstract nature of our model, although it would be very important when actual drugs are considered.) Among these six plots, the optimal combination generally does not align along the diagonal (i.e., combining two interventions at the same dose, as considered in Table 2), highlighting the need to refine dose combinations.

The dose–response surfaces and optimal dose in dual interventions in Figure 6 provide information about possible drug synergy. While there is no single quantitative definition of synergy, it broadly means that the combined effect of two drugs is larger than that expected from their sum [33,34]. If the optimal combined doses of drugs D and E are substantially smaller than either that of D or E alone, then there is a synergistic effect. On the other hand, if the optimal doses for drugs D+E are each close to that of drug D alone, then the addition of E is hardly useful, let alone synergistic. Unfortunately, the indications from our model suggest that the considered interventions are not especially synergistic in combination. According to Figure 6, some combinations look favorable if started at 1 mo, e.g., panels (c) and (e). However, the advantage disappears if started later at 10 mo. See panels (d) and (f) with belated treatments, where the optimal dosage is close to one of the axes ($V_{1}$ or $V_{1}^{\prime }$ monotherapy), indicating that adding a second intervention in $k_{2}$ is hardly useful in these cases. This shows that due consideration to dynamical analysis really is desirable in devising interventions. So far, clinical trials for single interventions of AD have only shown a modest slowdown in disease progression even in the best scenario. Our work here indicates that a cautious attitude should be held toward combinational interventions, at least in the context of the dynamical model of the mice data. Of course, AD pathogenesis is expected to be substantially different between sporadic human cases and genetically engineered mice [35]. To repeat a claim we have made before, it would be desirable to be able to develop a dynamical model based on human data.

## 4. Conclusions

We have proposed a kinetic model for a vicious cycle of mutual positive feedback to quantitatively describe reported data of Aβ accumulation in a mouse model. The data and kinetic model display a slow rise in Aβ level at young age, then a lengthy, huge jump (by at least two orders of magnitude), and a final slow upward drift towards saturation. A key finding is that the vicious feedback cycle is essential for reproducing the sigmoidal trend in time.

The model has been used to explore various intervention schemes: first single, then double interventions. The results show that interventions must be applied as early as possible. In fact, even strong single interventions fail to achieve the target Aβ reduction when implemented beyond a threshold of about 5 mo (out of a lifetime of 27 mo). Similar fairly sharp thresholds exist in intervention strength: an early but insufficiently strong intervention badly fails to suppress Aβ accumulation to the target level. Thus, both the strength and timing of interventions are critical. Between different single interventions, those interfering with either feedback cycle work better than directly accelerating the removal of Aβ or *X*, in terms of the minimum intervention strength required to achieve the same effect. These observations seem consistent with the very limited experimental and clinical observations in humans available so far.

We have also considered various combinations of two interventions. It is encouraging that two interventions of more modest strength can produce comparable results to either single intervention at high strength. On the other hand, there is no clear sign of synergy between two interventions. These results may have implications for real interventions in humans [36].

These findings reinforce widely held emerging views, especially the importance of early intervention and also somewhat the advantage of combination treatments. What this study highlights is the crucial role of the feedback vicious cycle, as revealed in the quantitative kinetic model with a dynamical systems approach. Analysis of the model highlights the importance of threshold behavior in the interventions. The model brings out the crucial importance of targeting the feedback; as well as the efficacy of targeting together both linear removal and nonlinear (feedback) production effects.

In our model, there is a single “X”-factor that interacts nonlinearly with Aβ in the vicious feedback cycle. The tau protein is an obvious candidate for *X*. In reality, of course, there may well be many *X* factors in complex interaction among each other and Aβ. It is important when relevant data become available to further clarify how these various feedback cycles interact. For example, do they act together as if there is effectively one big feedback loop, or is the situation essentially more complicated?

The positive feedback models proposed earlier by De Caluwé and Dupont [23], and more schematically by Burlando et al. [24], similar in some ways to ours, show bistability with a jump from a healthy to unhealthy state. This is not the picture seen in our model, which is more like a lifelong steady drift. We are focused on a transgenic mouse strain designed to demonstrate extreme Aβ accumulation, and the question of bistability might be different in an examination of analogous human data, if and when they become available.

Finally, as an extension of the work here, we are in the process of developing a related model based on available human data of Aβ plaques and tau tangles. Although the situation is much more complicated than transgenic mice, such models could help reveal the disease mechanisms.

## Figures and Tables

**Table 1:** Kinetic model of soluble Aβ42 in mouse brain with mutual positive feedback loops. Values indicated with an asterisk are fixed in the fitting.

$k_{2}$	mo^−1^	450 *
$V_{1}$	pg mg^−1^ mo^−1^	1.36 × 10^6^
$K_{1}$	–	10.7
A(1)	pg mg^−1^	34 *
$k_{2}^{\prime }$	mo^−1^	0.00168
$V_{1}^{\prime }$	mo^−1^	0.998
$K_{1}^{\prime }$	pg mg^−1^	134.2
X(1)	–	1 *
LogErr	–	0.540

**Table 2:** Required dose(s) for single and double interventions starting at t=1 to achieve A(27)≤100. Diagonal: single intervention. Off-diagonal: two interventions at the same dose (only half of them are shown due to symmetry).

	$k_{2}$	$V_{1}$	$V_{1}^{\prime }$
$k_{2}$	0.63	0.24	0.34
$V_{1}$	-	0.39	0.34
$V_{1}^{\prime }$	-	-	0.64

## Data Availability

The original contributions presented in this study are included in the article. Further inquiries can be directed to the corresponding author.
